# Supplementary material for: Do Framed Mental Health Messages on Social Media Influence University Students’ Motivation for Physical Activity?
Source: Int J Environ Res Public Health. 2021 Aug 17;18(16):8671. doi: 10.3390/ijerph18168671 (PMC8392601; doi:10.3390/ijerph18168671)
Supplement: Supplementary file 1 [file ijerph-18-08671-s001.zip › ijerph-1285211-supplementary.pdf]

Document S1: Pre- and post-intervention online questionnaire

Q1 Please provide consent if you understand all of the information on the previous page and would like to be included in this study.

- ◻ By ticking this box I DO give my consent for my anonymous responses to be used for internal and external reports and appropriate scientific outputs.
- ◻ By ticking this box I DO NOT give my consent for my anonymous responses to be used for internal and external reports and appropriate scientific outputs.

Q2 What is your full name?

Q3 If the name of your Facebook profile is different to your full name, please type your Facebook name here.

Q4 What is your email address?

Q5 What is your age?

Q6 What is your gender

- ◻ Male
- ◻ Female
- ◻ Other

Q6_a If you selected Other, please specify:

Q7 Please specify your ethnicity.

- ◻ Caucasian
- ◻ Latino/Hispanic
- ◻ Middle Eastern
- ◻ African
- ◻ Caribbean
- ◻ South Asian
- ◻ East Asian
- ◻ Mixed
- ◻ Other

Q7_a If you selected Other, please specify:

Q8 What is your occupation?

- ◻ Student
- ◻ Employed
- ◻ Other

Q8_a If you selected Other, please specify:

Q9 If you are a student, please provide the name of your university or college.

Q10 Please answer this question as truthfully as possible. Please read the following statements carefully and indicate to what extent you agree/disagree with each of the following items.

(Likert scale checklist option [strongly agree -> strongly disagree])

A. I feel motivated to be active (eg. any movement of your body which requires energy expenditure such as; active travel, occupational activity, carrying groceries)

B. I feel motivated to exercise (eg. activity associated with improving fitness such as going to the gym or playing in a sports team)

C. I feel motivated to be active with friends

D. I feel motivated to be active with family

E. I feel motivated to walk instead of driving/taking public transport

F. I do physical activity because I enjoy it

G. I am AWARE of the benefits associated with physical activity

H. I VALUE the benefits of physical activity

I. I am AWARE of the mental health benefits associated with physical activity

J. I VALUE the mental health benefits associated with physical activity

K. I am motivated to be active DUE TO the risks of inactivity

L. I am motivated to be active DUE TO the health benefits of physical activity

M. I don't see the point in doing physical activity

N. I do physical activity because other people say I should

O. I feel guilty when I don't do regular physical activity

P. I feel comfortable talking about mental health

The following questions have been taken from the International Physical Activity Questionnaire (IPAQ). The questions are about the time you spent being physically active in the **last 7 days.** They include questions about activities you do at work, as part of your house and yard work, to get from place to place, and in your spare time for recreation, exercise or sport.

In answering the following questions,

- **vigorous** physical activities refer to activities that take hard physical effort and make you breathe much harder that normal.
- **moderate** activities refer to activities that take moderate physical effort and make you breathe somewhat harder that normal.

Q11. During the last 7 days, on how many days did you do vigorous physical activities like heavy lifting, digging, aerobics, or fast bicycling? Think about only those physical activities that you did for at least 10 minutes at a time.

Q12. How much time in total did you usually spend on one of those days doing vigorous physical activities?

Q13. Again, think only about those physical activities that you did for at least 10 minutes at a time. During the last 7 days, on how many days did you do moderate physical activities like carrying light loads, bicycling at a regular pace, or doubles tennis? Do not include walking.

Q14. How much time in total did you usually spend on one of those days doing moderate physical activities?

Q15. During the last 7 days, on how many days did you walk for at least 10 minutes at a time? This includes walking at work and at home, walking to travel from place to place, and any other walking that you did solely for recreation, sport, exercise or leisure.

Q16. How much time in total did you usually spend walking on one of those days?

Q17. The last question is about the time you spent sitting on weekdays while at work, at home, while doing course work and during leisure time. This includes time spent sitting at a desk, visiting friends, reading traveling on a bus or sitting or lying down to watch television. During the last 7 days, how much time in total did you usually spend sitting on a week day? (Please answer in units of hours and minutes.)

Thank you for participating in this study!
